# Incidence and risk factors of falls in older people with chronic comorbidities in community: a cross-sectional study

**DOI:** 10.3389/fpubh.2025.1643699

**Published:** 2025-09-24

**Authors:** Siqin Liu, Yuanhua Si, Yiming Peng, Demin Wang, Xiaoli Yuan, Yan Peng

**Affiliations:** ^1^Department of Neurology, Affiliated Hospital of Zunyi Medical University, Zunyi, China; ^2^Department of Nursing, Affiliated Hospital of Zunyi Medical University, Zunyi, China

**Keywords:** comorbidity, health ecological model, falls, risk factors, older people

## Abstract

**Objective:**

To investigate the prevalence of falls among older adult individuals with comorbidities in community and to analyze the risk factors.

**Methods:**

Using multi-stage stratified random sampling method, a total of 886 older people aged over 60 years with comorbidities were selected from 10 communities as research subjects between April 2022 and December 2023. Data collection involved the use of a general information questionnaire, frail scale, self-rating anxiety scale, fall risk self-rating scale, standing up and walking timing test, and Berg Balance Scale. The patients were categorized into two groups based on fall occurrence, and χ^2^ test and logistic regression analysis were employed to identify the risk factors for falls.

**Results:**

The incidence of falls was 24.8% (220/886). The logistic regression analysis indicated that factors such as frailty, visual impairments, anxiety, fall risk score, physical exercise, social support, category of residence, and social interaction (OR = 0.535, 1.826, 4.284, 5.584, 1.965, 1.649, 2.308, 1.806; all *p* < 0.05) were significantly associated with falls in older people with comorbidities.

**Conclusion:**

The incidence of falls is higher among older people with comorbidities in the community, it is essential to prioritize older people with comorbidities who have limited daily social activities, insufficient social support, visual impairments, high fall risk scores, and impaired sit-stand and walking tests in order to prevent falls and promote healthy aging.

## 1 Introduction

The Outline of “Healthy China 2030” (1) emphasizes the importance of implementing health promotion initiatives for the older adult, highlighting their significant role in improving the overall health status, enhancing quality of life, and achieving healthy aging. Preventing falls is identified as a critical component of these health promotion efforts. According to reports, approximately 30% of individuals aged 65 years and older experience at least one fall annually, with the incidence rising to 50% among those aged 80 years and above. Among these falls, 20–30% result in injuries (2). Falls can lead to limitations in physical activity and functional capacity, reduced quality of life, increased economic burden, and may even result in disability or death, thereby imposing a significant burden on both families and society (3). Falls have become the number one cause of unintentional injury death among the older adult in China. Therefore, the health problems of the older adult caused by falls will become a serious problem in the national medical and health field, and the prevention and management of falls in the older adult has become a widespread concern. Multimorbidity means that the same patient suffers from 2 or more kinds of chronic diseases, long-term mental disorders or long-term infectious diseases (4). A meta-analysis of 193 chronic disease comorbidity studies from around the world showed that the final combined chronic disease comorbidity rate was 42.4% (5), according to the data of the National Health Statistical Yearbook, more than 3/4 of the older people in China suffer from 2 or more chronic diseases at the same time (6). At present, “multi-disease coexistence” has become an increasingly serious public health problem. In the future, it will gradually become a normal condition of health of older people in our country. Older people with comorbidities need to take a variety of oral drugs, such as antihypertensive drugs, hypoglycemic drugs, etc., which will cause adverse reactions such as postural hypotension and vertigo, thereby increasing the risk of falling (7, 8). In addition, physical function degradation, weakness, sarcopenia and cognitive impairment caused by aging and disease conditions greatly increase the risk of falling in patients with comorbidities. Therefore, it is important to understand the current situation of falls in older people with comorbidities, analyze the influencing factors, and formulate targeted interventions to reduce the risk.

Health ecology models integrate ecological theories with human health behaviors, emphasizing the influence of multiple interaction levels on health outcomes (9). This model comprises five distinct hierarchical layers. The core layer encompasses individual characteristics, such as gender, age, and susceptibility to diseases. The second layer includes behavioral traits of the individual, while the third layer reflects the interpersonal networks within society, such as family relationships, social interactions, and the status of children. The fourth layer pertains to living and working conditions, including economic status, occupational influences, and socioeconomic factors. The fifth layer addresses broader societal factors, such as political, economic, cultural, and policy-related environments. The model underscores that individual health outcomes are shaped by complex interactions among personal factors, social environments, healthcare policies, and cultural traditions. It is currently widely utilized in research related to disease prevention and health promotion for individuals with chronic conditions and specific vulnerable populations (10). This model enables a multi-dimensional, multi-level analysis of the factors contributing to falls among older adult individuals with comorbidities, thereby offering innovative insights and methodological approaches for fall prevention.

Although numerous studies have investigated falls and comorbidities among the older adult, comprehensive and systematic research examining the influencing factors of falls among older adults with comorbidities in community settings from the perspective of health ecology models remains limited. The absence of multidimensional and systematic analyses hinders in-depth exploration of the determinants of falls across multiple levels. Therefore, conducting such research holds significant practical value in guiding the development of targeted and effective fall prevention strategies, reducing the incidence of falls, and enhancing the quality of life of older people with comorbidities. Grounded in the health ecology model, this study aims to investigate the current status and associated factors of falls among older people with comorbidities, formulate evidence-based prevention strategies, provide a scientific foundation for policy-making, and support the realization of the goal of healthy aging.

## 2 Materials and methods

### 2.1 Study participants

The study enrolled older people who met the predefined inclusion and exclusion criteria and had received medical services at the community health service centers in Zunyi City, Guizhou Province, between April 2022 and December 2023. The selection process was conducted using the Community Residents’ Health Record Information System of Zunyi City, Guizhou Province, China, in conjunction with a multi-stage stratified sampling approach. In the first stage, Zunyi City was categorized into three major urban districts, from which two towns or streets were randomly selected in each. In the second stage, one to two township health centers or community health service centers were randomly chosen within each selected town or street. Finally, in the third stage, convenience sampling was applied to conduct face-to-face interviews with eligible older adult participants at the selected health service centers, and all participants have provided their informed consent.

#### 2.1.1 Inclusion criteria

(1) Presence of more than two concurrent chronic diseases, as determined by self-reported prevalence of 37 chronic conditions, including but not limited to hypertension, hyperlipidemia, diabetes, coronary heart disease, chronic heart failure, stroke, hemiplegia, angina pectoris, myocardial infarction, chronic bronchitis, emphysema, chronic obstructive pulmonary disease, asthma, arrhythmia, hypothyroidism, prostate disease, gout, cirrhosis, fatty liver, chronic enteritis, chronic hepatitis, peptic ulcer, chronic gastritis, chronic kidney disease, cholecystitis, osteoarthropathy, osteoporosis cervical/lumbar, spondylosis cataracts, rheumatoid arthritis, Parkinson’s disease, senile dementia, depression, anxiety, disorder malignant tumor and senile syndrome; (2) Age ≥ 60 years old; (3) Ability to communicate effectively and cooperate in completing the questionnaire and undergoing the walking function test; (4) Provision of informed consent and willingness to participate in the study; (5) Residency in the community for at least 6 months.

#### 2.1.2 Exclusion criteria

(1) Individuals who have been bedridden for an extended period or are suffering from severe physical illnesses; (2) individuals with psychiatric disorders or significant cognitive impairments that hinder effective communication; (3) individuals with severe visual, auditory, speech, or cognitive impairments, as well as other conditions that may prevent them from cooperating with the research team.

#### 2.1.3 Sample size

This study included a total of 30 independent variables. The recommended sample size was determined to be 15 times the number of variables, resulting in a target sample size of 450. To account for a potential 10% invalid response rate, the minimum required sample size was accordingly adjusted to 495. Ethical approval for this research was obtained from the hospital’s Ethics Committee (KLL2022-814).

### 2.2 Measures

The researchers designed a comprehensive data collection questionnaire that covers five key dimensions of HEM: personal traits, behavioral and lifestyle factors, interpersonal networks, working and living conditions, and the policy environment. Specifically, the questionnaire includes: (1) Personal characteristics, such as educational background, health status, gender, age, frailty, visual and auditory impairments; (2) behavioral traits and lifestyle factors, including quantity of medications, history of smoking and alcohol consumption, physical activity habits, psychological wellbeing (assessed through anxiety and depression indicators), fall risk assessment, walking function evaluation, and participation in routine physical examinations; (3) interpersonal network information, such as participation in social activities (e.g., playing board games, mahjong, traveling, dancing), marital status, and number of children; (4) working and living conditions, including per capita monthly household income, income sources, living arrangements, availability of social support, and housing type; and (5) policy-related factors, such as the type of medical insurance coverage.

#### 2.2.1 The frail scale

The concept of frailty was initially introduced by Fried et al. (11), and encompasses five components: self-reported exhaustion, reduced endurance, low levels of physical activity, muscular weakness, and unintentional weight loss. In this study, frailty was operationalized as a binary outcome. Each affirmative response to the listed criteria was assigned a score of 1, while a negative response received 0 points. The cumulative score ranged from 0 to 5. Individuals exhibiting three or more positive indicators were classified as frail, while those with one or two positive indicators were categorized as pre-frail. Based on the total score, participants were grouped into three categories: non-frail (0 points), pre-frail (1–2 points), and frail (3–5 points). The detailed assessment criteria are outlined as follows:

1 Have you felt tired most or all of the past 4 weeks?
2 If there is no midway break or climbing one floor with the assistance of walking aids, do you feel any difficulty?
3 Do you feel any difficulty walking 100 meters distance with the assistance of helpless walking equipment?
4 Do you suffer from 5 or more diseases?
5 Have you lost more than 5% of your weight in the past year?

#### 2.2.2 Self-rating anxiety scale

The Zung Anxiety Scale, developed by Zung in 1984 (12), utilizes a 4-point scale to assess an individual’s subjective feelings of anxiety through 20 items. The Cronbach’s *α* coefficient of the scale was calculated at 0.81. Each item is scored from “never or occasionally” to “always,” with points ranging from 1 to 4. The total coarse score for each item is multiplied by 1.25 to obtain the standard score. A standard score below <50 indicates no anxiety, while scores between 50 and 59 are classified as mild anxiety, scores between 60 and 69 as moderate anxiety, and scores ≥70 as severe anxiety.

#### 2.2.3 Geriatric depression scale-15 (GDS-15)

The GDS-15 is specifically designed to evaluate depressive symptoms in older age groups (13), this scale comprises 15 items, each scored with “yes” or “no,” where “yes” corresponds to 1 point and “no” corresponds to 0 points. Items 1, 5, 7, and 11 are reverse-scored such that a response of “no” earns 1 point; the total score ranges from 0 to 15.demonstrating a Cronbach’s *α* coefficient of 0.82 (14). Scores of ≤4 indicate normal status; scores of 5–9 suggest mild depression; while scores of10–15 indicate moderate to severe depression.

#### 2.2.4 The self-rated fall risk scale

The self- rated fall risk scale among the older adult, developed by the Centers for Disease Control and Prevention (CDC) in the United States (15), is a tool designed to enable older adult individuals to evaluate their own risk of falling. The scale comprises 12 questions that require a “yes” or “no” response. For the first two items, a “yes” answer is assigned 2 points, while for the remaining 10 items, a “yes” answer is awarded 1 point and a “no” answer receives 0 points. The maximum total score is 14 points. A higher score indicates a greater likelihood of fall risk. Individuals scoring 4 or higher are considered to be in the high-risk category for falls.

#### 2.2.5 Standing up and walking timing test

The standing and walking timing test require the subject to remain seated in a chair with their back resting against the backrest and hands placed on the armrests. Upon receiving the “go” command, the subject is instructed to stand up from the chair, walk forward as quickly as possible for a distance of 3 meters, reach a designated marked line, and then return to a seated position in the original chair, the total duration of the task is recorded in seconds (16). According to data from the CDC of the United States, older adult individuals who require 12 s or more to complete this test may be at an increased risk of falling (15). Therefore, this study defines successful completion of the test as achieving a time of <12 s.

#### 2.2.6 Berg balance scale

The Berg Balance Scale (BBS) (17) evaluates a series of functional tasks, including sit-to-stand, unsupported standing, unsupported sitting, stand-to-sit, transfers, standing with eyes closed, standing with both feet together, reaching forward with the arms, bending down to pick up objects, turning the head to look behind, turning around in a full circle, stepping onto an alternate foot stool, and standing in a straight line with one foot directly in front of the other. Each task is scored on a 5-point scale ranging from 0 to 4, depending on the individual’s ability to perform the task: a score of 0 indicates inability to complete the task, while a score of 4 reflects normal performance. The total score ranges from 0 to 56. A score between 0 and 20 indicates poor balance function; a score between 21 and 40 suggests moderate balance ability; a score below 40 indicates a high risk of falling; and a score between 41 and 56 reflects good balance function. In this study, impaired balance function was defined as a score below 40, whereas a score above 40 was considered indicative of good balance function.

### 2.3 Research methods

Prior to the initiation of the investigation, the research team contacted the designated representatives of each community to obtain their consent and support. Subsequently, five trained and certified assessors conducted questionnaire surveys at the community activity centers. Prior to data collection, the purpose and methodology of the study were thoroughly explained to older people with comorbidities or their family members who met the inclusion and exclusion criteria, and written informed consent was obtained. During the survey process, standardized instructions were used to ask questions in a systematic manner. Assessors provided clear, detailed, and unambiguous explanations in response to participants’ inquiries. For participants who encountered difficulties in completing the questionnaire, assessors recorded responses based on the participants’ verbal answers in an objective and accurate manner. A total of 898 questionnaires were distributed. After excluding 12 invalid questionnaires due to incomplete entries, incorrect formatting, repetitive responses, or logical inconsistencies, 886 valid responses remained, resulting in an effective response rate of 98.66%.

### 2.4 Statistical analyses

The data were input using Excel software and verified through a dual parallel entry approach. Statistical analysis and description were conducted using the SPSS 26.0 (18) software package. Descriptive statistics were presented as frequency (n) and percentage (%), while inferential statistics involved the use of the chi-square (*χ*^2^) test. Furthermore, a multivariate logistic regression model was employed to examine the influencing factors associated with falls in older people with comorbidities, with a significance level set at *α* = 0.05.

## 3 Results

### 3.1 Fall status of older people with comorbidities in community

**Figure 1:**
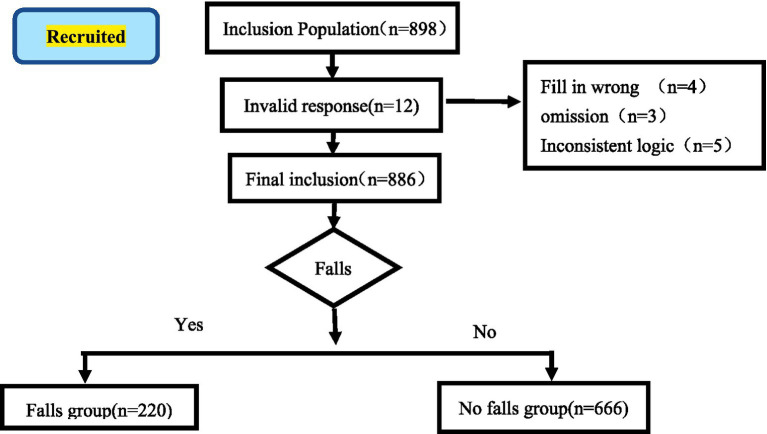
Recruitment of research participants.

A total of 898 older people with comorbidities were enrolled in this study (see Figure 1 for the participant recruitment process). Among them, 12 provided invalid responses, and 886 provided valid responses. The mean age of the participants was 73.06 ± 7.60 years, ranging from 60 to 90 years. The gender distribution consisted of 440 males (49.66%) and 446 females (50.34%). The incidence of falls was observed in 24.83% (220/886) of the participants, and further demographic characteristics are summarized in Table 1.

**Table 1:** Analysis of factors affecting falls in older people with comorbidities in community.

Health ecological hierarchy	Variable	Group	No falls	Falls	X^2^ value	*p*-value
			*n* = 666	*n* = 220		
Personal trait	Age	60–69	284 (32.1)	90 (10.2)	1.118	0.572
		70–79	221 (24.9)	69 (7.8)		
		≥80	161 (18.2)	61 (6.9)		
	Gender	Male	332 (37.5)	108 (12.2)	0.038	0.845
		Female	334 (37.7)	112 (12.6)		
	Education	Below primary school	311 (35.1)	116 (13.1)	3.938	0.268
		Primary school	155 (17.5)	48 (5.4)		
		Junior high school	104 (11.7)	24 (2.7)		
		High school/technical secondary school and above	96 (10.8)	32 (3.6)		
	Health status	Good	117 (13.2)	33 (3.7)	8.761	0.013
		General	434 (49.0)	129 (14.6)		
		Poor	115 (13.0)	58 (6.5)		
	Frailty	No frailty	357 (40.3)	94 (10.6)	7.974	0.019
		Pre-frailty	180 (20.3)	71 (8.0)		
		Frailty	129 (14.6)	55 (6.2)		
	Visual difficulties	No	411 (46.4)	92 (10.4)	26.668	<0.001
		Yes	255 (28.8)	128 (14.4)		
	Hearing difficulty	No	548 (61.9)	163 (18.4)	7.001	0.008
		Yes	118 (13.3)	57 (6.4)		
Behavior and lifestyle	Drinking history	No	561 (63.3)	190 (21.4)	0.581	0.446
		Yes	105 (11.9)	30 (3.4)		
	Smoking history	No	528 (59.6)	181 (20.4)	0.927	0.336
		Yes	138 (15.6)	39 (4.4)		
	Quantity of medications	0	133 (15.0)	40 (4.5)	0.699	0.705
		1–2	339 (38.3)	110 (12.4)		
		≥3	194 (21.9)	70 (7.9)		
	Exercise	Yes	488 (55.1)	109 (12.3)	42.362	<0.001
		No	178 (20.1)	111 (12.5)		
	Depressed	No depressed	434 (49.0)	146 (16.5)	4.101	0.129
		Mild depressed	186 (21.0)	51 (5.8)		
		Moderate to severe depressed	46 (5.2)	23 (2.6)		
	Anxiety	No anxiety	635 (71.7)	177 (20.0)	43.201	<0.001
		Mild anxiety	31 (3.5)	41 (4.6)		
		Moderate to severe anxiety	0 (0.0)	2 (0.2)		
	Fall risk	≥4 score	78 (8.8)	77 (8.7)	62.138	<0.001
		<4 score	588 (66.4)	143 (16.1)		
	Balance test	Good balance function	424 (47.9)	107 (12.1)	15.551	<0.001
		Impaired balance function	242 (27.3)	113 (12.8)		
	Walk-sit test	≥12 s	214 (24.2)	97 (10.9)	10.382	0.001
		<12 s	452 (51.0)	123 (13.9)		
Interpersonal network	Marital status	At marriage	549 (62.0)	167 (18.8)	4.538	0.033
		Nonexistent marriage	117 (13.2)	53 (6.0)		
	Social activities	No	207 (23.4)	120 (13.5)	39.101	<0.001
		Yes	459 (51.8)	100 (11.3)		
	Number of children	0	11 (1.2)	4 (0.5)	3.496	0.321
		1	80 (9.0)	29 (3.3)		
		3	186 (21.0)	74 (8.4)		
		≥3	389 (43.9)	113 (12.8)		
Living and working conditions	Resident manner	Live alone	35 (4.0)	17 (1.9)	1.829	0.176
		Live with people	631 (71.2)	203 (22.9)		
	Place of Residence	Rural	370 (41.8)	99 (11.2)	7.396	0.007
		Urban	296 (33.4)	121 (13.7)		
	Monthly income	<2 k	313 (35.7)	108 (12.2)	1.184	0.668
		>2 k	344 (39.3)	111 (12.7)		
	Source of income	Labor income	127 (14.3)	41 (4.6)	1.418	0.701
		Child giving	273 (30.8)	96 (10.8)		
		Salary/pension	237 (26.7)	77 (8.7)		
		Others	29 (3.3)	6 (0.7)		
	Social support	Sufficient	518 (58.5)	130 (14.7)	29.394	<0.001
		Insufficient	148 (16.7)	90 (10.2)		
	Routine physical examination	No	335 (37.8)	86 (9.7)	8.332	0.004
		Yes	331 (37.4)	134 (15.1)		
Policy environment	Medical insurance type	Medical insurance	631 (71.2)	215 (24.3)	3.412	0.065
		Self-financing	35 (4.0)	5 (0.6)		

### 3.2 Analysis of factors affecting falls of older people with comorbidities in community

The results of the univariate analysis indicated statistically significant differences among groups across 14 factors, including health status, frailty, vision impairment, hearing impairment, fall risk, physical activity, social engagement, sit-stand/walk test performance, balance test outcomes, and participation in regular physical examinations (*p* < 0.05), as presented in Table 1.

### 3.3 Multivariate analysis of falls among older people with comorbidities in community

Whether older adult individuals with comorbidities in the community had experienced falls was used as the dependent variable. An initial univariate analysis was conducted on the independent variables that demonstrated statistical significance within the five-level health ecological model (variable coding is provided in Table 2). These significant variables were then entered into a logistic regression model using a stepwise forward selection procedure. The results revealed that residential status, participation in social activities, level of social support, visual impairment, fall risk score, frailty, engagement in physical exercise, and anxiety were statistically significant predictors of falls among older adult individuals with comorbidities (all *p* < 0.05), as presented in Table 3.

**Table 2:** Logistic regression analysis of variable assignment.

Variable	Variable assignment
Dependent variable (Y)	0 = No fall; 1 = Fall
Independent variable (X)	
X_1_: Place of Residence	1 = Urban; 2 = Rura
X_2_: Social support	1 = Sufficient; 2 = Insufficient
X_3_: Social activities	1 = Yes; 2 = No
X_4_: Visual difficulties	1 = No; 2 = Yes
X_5_: Fall risk	1 = <4 score; 2 = ≥4 score
X_6_: Frailty	1 = No frailty; 2 = Pre-frailty; 3 = Frailty
X_7_: Exercise	1 = Yes; 2 = No
X_8_: Anxiety	1 = No anxiety; 2 = Mild anxiety; 3 = Moderate to severe anxiety

**Table 3:** Logistic multivariate regression analysis of falls in older people with comorbidities in community.

Health ecological hierarchy	Variable	Model 1 OR (95%CI)	Model 2 OR (95%CI)	Model 3 OR (95%CI)	Model 4 OR (95%CI)
Personal characteristics	Frailty	1.597 (1.075–2.370) *	0.515 (0.3–0.885) *	0.527 (0.304–0.914) *	0.535 (0.307–0.932)*
	Visual difficulties	2.258 (1.653–3.085) **	2.081 (1.483–2.918) **	1.84 (1.302–2.600) **	1.826 (1.307–2.584) **
Behavior feature	Anxiety		4.403 (2.549–7.605) **	4.243 (2.429–7.414) **	4.284 (2.443–7.511)**
	Fall risk		5.443 (3.322–8.920) **	5.556 (3.358–9.193) **	5.584 (3.361–9.279) **
	Exercise		2.441 (1.732–3.440) **	2.603 (1.782–3.803) **	1.965 (1.283–3.011) **
Living condition	Social support			1.829 (1.243–2.691) **	1.649 (1.112–2.446) *
	Place of Residence			2.222 (1.536–3.214) **	2.308 (1.59–3.350)**
Interpersonal network	Social activities				1.806 (1.189–2.744) *
Constant		0.177	0.008	0.001	0.001
Pseudo-R^2^		0.038	0.156	0.179	0.186
Prediction ratio		0.628	0.75	0.774	0.78

① OR: Risk ratio; 95%CI: 95% confidence intervals; ② **p < 0.001, *p < 0.05; ③ The prediction ratio is the percentage that the model predicts accurately.

## 4 Discussion

The increasing aging of the population has led to a rise in age-related health problems, with 23.8% of the older adult suffering from two or more chronic diseases. Globally, approximately 28 to 35% of individuals aged 65 and older experience falls annually, and the incidence increases with age. During the 14th Five-Year Plan period, China’s aging population is expected to grow at an accelerated pace, with the number of people aged 60 and above surpassing 300 million, marking China’s transition from mild to moderate aging. With this acceleration, the phenomenon of chronic disease comorbidity is becoming increasingly prevalent. According to research findings, the prevalence of chronic disease comorbidity among the older adult is as high as 65.16% (19), indicating a significant public health concern. A separate study (20) reported that the fall incidence rate among the older adult is 15.8%. In this study, the fall incidence rate among community-dwelling older adult with comorbidities was found to be 24.83%, which is consistent with the results reported by Zhang et al. (21). These findings suggest that older adult individuals with comorbidities are more prone to falls compared to their healthier counterparts. One possible explanation is that chronic diseases, as long-term conditions, represent a significant risk factor for falls. Moreover, due to the progressive nature of aging and comorbidities, many older adult individuals experience declines in physical function, cognitive abilities, motor coordination, vision, and overall physiological function, along with slower reaction times. These factors contribute to increased vulnerability and reduced stability, potentially explaining the higher fall rates among this population. Falls among the older adult can lead to severe consequences, including functional impairment, physical disability, and even mortality, significantly affecting their quality of life. According to the World Health Organization (WHO), 28 to 35% of individuals over the age of 65 fall annually, with 4 to 15% of these falls resulting in major injuries and 23 to 40% of injury-related deaths attributed to falls (22). Domestic studies further indicate that among individuals aged 60 and above, falls are the leading cause of unintentional injuries, accounting for approximately 52.81% of all such incidents (21). Therefore, the older adult represent a high-risk group for falls. Effectively preventing falls among older adult individuals with comorbidities is crucial for alleviating family caregiving burdens and improving patients’ quality of life. Fall risk assessment serves as the foundational step in fall prevention, highlighting the importance of establishing standardized identification and management protocols for fall risks among older people with comorbidities. However, current research primarily focuses on the status and assessment tools related to falls among the general older population and those with chronic diseases, with limited attention given to fall identification and management strategies specifically for older adult individuals with comorbidities. It is recommended that community healthcare systems prioritize fall prevention among older people with comorbidities by developing targeted risk identification and management procedures. The STEADI (Stopping Older adult Accidents, Deaths, and Injuries) toolkit has demonstrated effectiveness in reducing falls among the older adult (15). In the future, communities may adopt the STEADI assessment tools to systematically evaluate fall risks among older people with comorbidities. Concurrently, proactive disease management, tailored to individual characteristics, should be implemented to identify and address specific risk factors. Additionally, health education and lifestyle guidance should be provided to older adult individuals with comorbidities who are at risk of falling, with the aim of reducing fall incidence.

Personal traits are a primary factor influencing the occurrence of falls, visually impaired individuals exhibit reduced sensitivity to environmental changes, limited access to updated information during such changes, and compromised visual acuity, which may impair posture balance and increase the risk of falls (23). Studies have shown a significant correlation between overall visual function in the older adult and their balance and mobility. Vision plays a crucial role in maintaining postural stability, locomotion, and fall prevention (24). Our research indicates that visual impairment is an independent risk factor for falls among older people with comorbidities (OR = 1.826, 95% CI: 1.307–2.584), a finding consistent with previous studies (25). Therefore, it is essential to prioritize the assessment of visual function in older adult patients. Furthermore, tailored fall prevention and management strategies should be implemented for visually impaired individuals, such as actively treating underlying conditions to improve visual function, enhancing health education, and providing psychological counseling to reduce fall risk. However, it should be noted that current domestic and international fall prevention guidelines offer limited interventions specifically targeting visually impaired individuals. There is a relative lack of fall prevention strategies tailored to this population. Hence, future development of targeted fall prevention measures for visually impaired patients is necessary, which would have significant implications for improving their quality of life and enhancing safety management.

This study revealed that the fall risk score was significantly associated with the likelihood of patients experiencing falls (OR = 5.584, 95% CI: 3.485–6.876), a finding consistent with previous research (26). There are several underlying reasons for this association. First, older people with comorbidities often exhibit long-standing risk factors for falls, thereby increasing their susceptibility to falling (27). Second, such patients typically experience diminished neuromuscular control and reduced balance capacity. Combined with age-related declines in muscle strength and overall body stability, these impairments make it difficult for them to maintain postural control in response to sudden stimuli, ultimately increasing the risk of falls. The ability to sit, stand, and walk serves as a key indicator of lower limb motor function in older adults, while balance is essential for maintaining posture and stability. Consequently, gait and balance abilities are closely linked to fall occurrence. Wang Li et al. (28) reported that older adult individuals with impaired walking and balance performance are more prone to falls, a result corroborated by this study (*p* < 0.05). Although many physiological conditions are irreversible, behavioral traits and lifestyle habits are modifiable. Therefore, healthcare professionals can develop individualized and practical intervention strategies tailored to each patient’s specific condition to enhance core functional abilities such as sitting, walking, and balancing. Targeted strength and balance training for the lower limbs can improve reaction time and postural stability, thereby reducing fall risk, preventing falls, and promoting healthy aging.

This study demonstrates that social activities serve as a protective factor against falls in older people with comorbidities (OR = 1.806, 95% CI: 1.189–2.744). In other words, social participation can effectively reduce the risk of falls in this population, which aligns with findings from previous research (29). Although this conclusion may appear to contradict traditional beliefs—wherein it was commonly thought that older adult individuals should remain at home and rest more to minimize fall risks—lack of social participation is increasingly viewed as a deficiency in access to essential resources that fulfill basic social needs. Meeting these social needs is crucial for fostering self-efficacy. Consequently, insufficient social engagement may heighten the likelihood of falls among the older adult (29). For older people with comorbidities who exhibit limited social participation, it is particularly important to focus on enhancing their sense of self-efficacy and encouraging them to engage in suitable social activities based on their individual physical conditions. Meaningful social activities not only promote appropriate physical exercise, improve physical fitness, enhance sleep quality, and overall health, but also support psychological wellbeing and enrich daily life experiences. Therefore, community or village committees should consider organizing structured social activities for the older adult, such as singing, dancing, chess-playing, and reading sessions, to promote both physical activity and cognitive engagement. Healthcare professionals should encourage older people with comorbidities to actively participate in such activities and develop progressive social engagement plans tailored to each patient’s age, physical condition, and prior exercise habits. These efforts can help reduce social isolation and emotional loneliness, enhance overall health, and ultimately prevent falls.

This study revealed that adequate social support serves as a protective factor against falls among older people with comorbidities (OR = 1.649, 95% CI: 1.112–2.446), which aligns with findings from prior research (29). Social support plays a crucial role in reducing the risk of falls in this population. Potential explanations for this association include the following: older people with multiple chronic conditions often require substantial assistance in daily living and emotional support. Strong social support enables these individuals to access necessary help through their social networks, compensating for functional limitations caused by aging and illness. It also facilitates better access to healthcare services and improves adherence to medication regimens. Moreover, the effectiveness of fall prevention programs is closely linked to the level of social support available. Greater social support is associated with increased patient engagement and confidence in fall prevention initiatives. Therefore, it is recommended that society at large strengthen its support for older adult individuals with comorbidities by integrating community and family resources to provide comprehensive life, medical, and emotional assistance. Healthcare professionals should encourage patients to build and utilize effective social support systems, reduce psychological stress, foster confidence in fall prevention, and promote self-management behaviors. These efforts can ultimately help reduce the incidence of falls and mitigate their adverse consequences.

Falls among the older adult are influenced by their living environment (30). At the environmental level, this study found that the incidence of falls among older adult individuals with comorbidities in rural areas (29.02%) was significantly higher than that in urban areas (21.11%) (*p* < 0.05), which aligns with findings from previous studies (31). This disparity may be attributed to differences in economic income, access to healthcare, the degree of community adaptation for the older adult, and overall living conditions between urban and rural areas. Given that most older adult individuals in rural areas lack a stable income and have engaged in long-term agricultural labor, their skeletal muscle function and balance capacity may deteriorate more rapidly. Additionally, rural communities generally place less emphasis on optimizing home-based older adult care environments, face relatively limited medical resources, and exhibit insufficient awareness of fall prevention strategies, all of which contribute to an increased risk of falls. Therefore, it is crucial to prioritize education and guidance on fall self-management for individuals with low educational attainment, limited income, and those residing in rural areas. Through targeted educational interventions, the older adult can be empowered to identify and mitigate environmental risk factors, thereby reducing the likelihood of falls.

**Figure 2:**
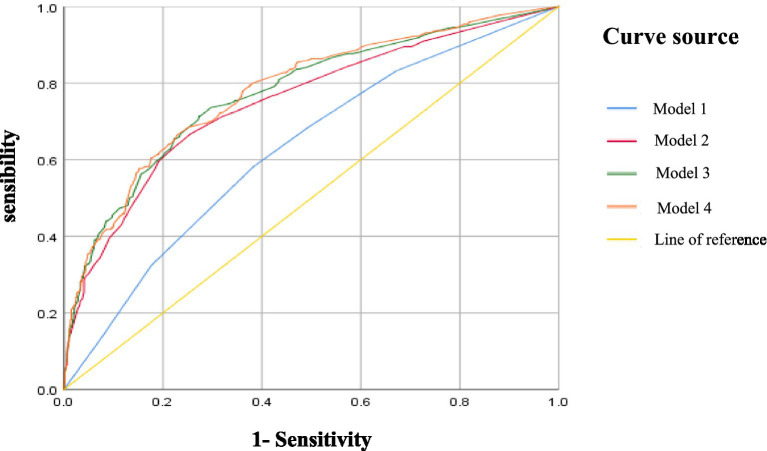
ROC curve of fall prediction model for older people with comorbidities in community.

Both Table 3 and Figure 2 illustrate that each dimension of the health ecology model contributes to explaining the occurrence of falls among older people with comorbidities in community settings. The regression results indicate that as variables representing each level of the health ecology model are progressively incorporated into the analysis, the model’s explanatory power increases, and its predictive accuracy improves accordingly. This suggests that no single intrinsic factor alone can fully account for the risk of falls in older adult individuals with multiple chronic conditions. Rather, this risk is shaped by a combination of individual characteristics, behavioral patterns, interpersonal interactions, and environmental conditions, reflecting the interplay within a broader health ecology. Therefore, prior to developing fall prevention strategies and interventions, a comprehensive individual assessment should be conducted. By adopting a health ecology-based approach, healthcare professionals can evaluate patients’ fall risks from multiple perspectives—including personal attributes, family and social support, physical activity, and balance capacity—and implement early, targeted nursing interventions to address the issue of falls among older people with comorbidities.

### 4.1 Strengths and limitations

The strength of this study lies in its comprehensive approach based on the health ecology model, which covers multiple levels of factors—including individual, interpersonal, community, and policy-related aspects—thereby overcoming the limitations of traditional single-factor analyses. This multi-level perspective enables a more systematic and in-depth examination of fall-related issues among older adult individuals with comorbidities in community settings, thereby providing a solid foundation for the development of comprehensive intervention strategies. In terms of sampling, a multi-stage stratified sampling method was employed. This approach ensures that samples are appropriately distributed across different strata, effectively minimizing sampling bias and enhancing sample representativeness. As a result, the findings are more reflective of the actual situation and influencing factors of falls among older adult individuals with comorbidities across communities with varying characteristics, thereby strengthening the reliability of the research conclusions. However, this study also has several limitations. First, although the study involved multiple communities, the sample was limited to patients from Zunyi City, Guizhou Province, China, which imposes certain geographical constraints. Future research should expand the sample to include regions with varying levels of economic development and diverse cultural backgrounds in order to enhance the generalizability of the findings. Second, the cross-sectional design limits the ability to establish causal relationships between the studied factors and falls, as it can only identify correlations. Prospective cohort studies are recommended in future research to track changes in these factors over time and their impact on fall occurrences, thereby clarifying the underlying causal mechanisms. Third, some data were collected through self-reports from participants, which may introduce recall bias. To address this limitation, future studies could incorporate objective measurement methods, such as using wearable devices to monitor daily activities and fall incidents, thereby improving data accuracy and reliability.

## 5 Conclusion

Our research findings indicate that the incidence of falls among older adult individuals with comorbidities in the community is notably high. This phenomenon is influenced by a combination of factors, including personal attributes, behavioral patterns, social interactions, and living and working environments, and is ultimately shaped by the interplay within the broader health ecological system. Therefore, in addition to maintaining the physical and mental wellbeing of the older adult, it is equally important to address external factors that contribute to fall risk. Future community interventions should focus on closely monitoring the health status of older people with comorbidities, strengthening their social support networks, promoting active social engagement, improving their living conditions, implementing targeted balance training programs, and ultimately reducing the occurrence of fall-related incidents.

## Data Availability

The raw data supporting the conclusions of this article will be made available by the authors, without undue reservation.
