# Supplementary material for: Incidence and risk factors of falls in older people with chronic comorbidities in community: a cross-sectional study
Source: Front Public Health. 2025 Sep 24;13:1643699. doi: 10.3389/fpubh.2025.1643699 (PMC12504252; doi:10.3389/fpubh.2025.1643699)
Supplement: Supplementary file 1 [file Table_1.docx]

Dear senior friends:

Hello! We sincerely invite you to participate in the investigation and research of the national key research and development project "Active Health and Aging Technology Response Project". Your information will only be used for project research and there will be no privacy leakage. You just need to follow my instructions to complete this health assessment. There is no right or wrong answer to all the questions. Just complete it truthfully according to your actual situation. Thank you very much for your cooperation!

**Questionnaire on Elderly Capacity Assessment**

**Assessor information**

**Name：**________________ **Assess date**：____________

**Interviewee information:**

**Personal trait:**

**interviewee's name：______________ Gender:** ☐ male ☐ female **Age**________________-

**The residential address:** _______district, _______community

**Height:** ______ cm. **Weight:** ______kg

**Educational background:** ☐ primary school or below ☐ primary school ☐ junior high school ☐ senior high school or above

**Health status:** ☐ poor ☐ average ☐ good

**Frailty:** ☐No frailty ☐Frailty ☐Pre-frailty

**Visual difficulties:** ☐ Yes ☐ No

**Hearing difficulties:** ☐ Yes ☐ No

Note: Frailty was evaluated using the Frailty Screening Scale.

**Behavior and lifestyle**

**Drinking situation:** ☐ always drink ☐ never drink

☐ once drank, has quit (please describe specifically, how many two times:____ how many years:____)

**Smoking status:** ☐ always smoked ☐ never smoked

☐ once smoked, quit (please describe specifically, how many cigarettes/times:______ how many years:______)

**Long-term Quantity of medications:** ☐ none ☐1 type ☐2 types ☐3 types ☐4 types or more

**Take exercise:** ☐ Yes ☐ No

Note: Depressed, Anxiety, Fall risk, Balance test, Walk-sit The tests were evaluated respectively using the Self-Rating Anxiety Scale, GDS-15, Self-Rating Fall Risk Scale, Stand-up and Walk Timing test, and Berg Balance Scale.

**Interpersonal network:**

**Marriage:** ☐ married ☐ unmarried (unmarried, divorced, widowed)

**Social activities** (e.g., playing board games, mahjong, traveling, dancing): ☐ Yes ☐ No

**Number of children:** ☐ no children ☐1 children ☐2 children ☐3 or more

**Living and working conditions**

**Resident manner：**☐ live with others ☐ live alone

**Residence category:** ☐ urban ☐ rural

**Monthly income：**☐ <2k ☐ >2k

**Source of income:** ☐ Labor income ☐Child giving ☐Salary/pension ☐ Others

**Social support:** ☐ Insufficient ☐ Sufficient

**Routine physical examination:** ☐ Yes ☐ No

Policy environment

**Medical insurance type:** ☐Medical insurance ☐Self-financing
